# Auxin-induced *WUS* expression is essential for embryonic stem cell renewal during somatic embryogenesis in Arabidopsis

**DOI:** 10.1111/j.1365-313X.2009.03880.x

**Published:** 2009-05-07

**Authors:** Ying H Su, Xiang Y Zhao, Yu B Liu, Chuan L Zhang, Sharman D O’Neill, Xian S Zhang

**Affiliations:** 1 State Key Laboratory of Crop Biology, College of Life Sciences, Shandong Agricultural UniversityTaian, Shandong 271018, China; 2 Department of Plant Biology, College of Biological Sciences, University of CaliforniaDavis, CA 95616, USA

**Keywords:** *WUS* expression, auxin, stem cells, shoot apical meristem, somatic embryogenesis, Arabidopsis

## Abstract

Somatic embryogenesis requires auxin and establishment of the shoot apical meristem (SAM). *WUSCHEL* (*WUS*) is critical for stem cell fate determination in the SAM of higher plants. However, regulation of *WUS* expression by auxin during somatic embryogenesis is poorly understood. Here, we show that expression of several regulatory genes important in zygotic embryogenesis were up-regulated during somatic embryogenesis of Arabidopsis. Interestingly, *WUS* expression was induced within the embryonic callus at a time when somatic embryos could not be identified morphologically or molecularly. Correct*WUS* expression, regulated by a defined critical level of exogenous auxin, is essential for somatic embryo induction. Furthermore, it was found that auxin gradients were established in specific regions that could then give rise to somatic embryos. The establishment of auxin gradients was correlated with the induced *WUS* expression. Moreover, the auxin gradients appear to activate PIN1 polar localization within the embryonic callus. Polarized PIN1 is probably responsible for the observed polar auxin transport and auxin accumulation in the SAM and somatic embryo. Suppression of *WUS* and *PIN1* indicated that both genes are necessary for embryo induction through their regulation of downstream gene expression. Our results reveal that establishment of auxin gradients and PIN1-mediated polar auxin transport are essential for *WUS* induction and somatic embryogenesis. This study sheds new light on how auxin regulates stem cell formation during somatic embryogenesis.

## Introduction

Somatic embryogenesis has been recognized as an important way for regenerating entire plants, and as a potential model for analyzing the regulation of gene expression during plant embryo development (Meinke, 1991; Birnbaum and Alvarado, 2008). The original description of somatic embryogenesis in plants stems from observations of carrot cell cultures (Steward *et al.*, 1958). Somatic embryos of carrot were induced from single cells isolated from cultured callus, and this induction requires hormones or plant growth regulators.

The most critical event in embryo development appears to be the formation of the meristem (Meinke, 1991). Stem cells within the meristem enable plants to grow and produce new organs (Scheres, 2007), and their identity and proliferation are maintained in part by signals that these cells receive from the local environment. In the shoot apical meristem (SAM) of Arabidopsis, *WUSCHEL* (*WUS*) is a critical regulator, and encodes a homeodomain protein that is required for stem cell formation and maintenance (Laux *et al.*, 1996). In *wus* mutants, plants are unable to maintain a pool of undifferentiated stem cells (Laux *et al.*, 1996). *WUS* expression is confined to a small group of cells within the central zone, referred to as the SAM organizing center or stem cell niche, which is located immediately below the apical layer of active stem cells (Mayer *et al.*, 1998; Schoof *et al.*, 2000). WUS activity results in signaling to the overlaying stem cells, inducing *CLAVATA3* (*CLV3*) and its secreted protein, CLV3, which is thought to act as the ligand for the CLV1/CLV2 receptor complex that limits the area of *WUS* expression in the underlying organizing center. The CLV3/WUS negative feedback loop ensures homeostasis of the SAM by regulating the number of stem cells present in the central zone (Fletcher *et al.*, 1999; Brand *et al.*, 2000; Schoof *et al.*, 2000; Rojo *et al.*, 2002; Lenhard and Laux, 2003). During zygotic embryogenesis, *WUS* is initially expressed in the 16-cell pro-embryo, preceding meristem development within the region that gives rise to stem cells and the embryonic shoot (Mayer *et al.*, 1998).

Genetic analysis has indicated that polar auxin transport operates not only in inflorescence development but also in embryogenesis (Okada *et al.*, 1991; Liu *et al.*, 1993). Treatment of early globular to heart-shaped stage embryos of Indian mustard (*Brassica juncea*) with polar auxin transport inhibitors resulted in an abnormal partial fusion of cotyledons. *PIN-FORMED* (*PIN*) genes encode transmembrane proteins that are involved in auxin transport in Arabidopsis (Wiśniewska *et al.*, 2006). PIN1, a component of auxin efflux carriers, is required for the initiation and maintenance of an auxin gradient in plant tissues (Friml *et al.*, 2003). During early embryogenesis, it was shown that efflux-dependent auxin gradients play an important role in apical–basal axis establishment (Friml *et al.*, 2003).

The capacity for somatic embryogenesis was examined in *leafy cotyledon* (*lec*) mutants of Arabidopsis (Gaj *et al.*, 2005). The *lec* mutants are strongly impaired in terms of their embryonic response, and *LEC* genes probably function downstream in auxin-induced somatic embryogenesis (Stone *et al.*, 2001). Although the zygotic embryo originates from the zygote within the embryo sac, and the somatic embryo can be derived from either a single cell or be of multi-cellular origin depending on the culture conditions, both types of embryo progress through similar developmental stages from the globular stage to the torpedo stage, and share many morphological features during this development (Meinke, 1991; Zimmerman, 1993).

Stem cells must be formed within the SAM during somatic embryogenesis. The *WUS* gene, acting as a transcriptional regulator, is critical for the regulation of stem cell fate in plants (Mayer *et al.*, 1998; Gallois *et al.*, 2004). Although hormones are required for shoot regeneration from callus, somatic embryogenesis can be induced from callus by the removal of auxins (Zimmerman, 1993; Gordon *et al.*, 2007). Thus, in this study, we address the question of how auxin regulates *WUS* expression during somatic embryogenesis. We show that auxin gradients are established in specific regions of embryonic callus, and that *WUS* induction is correlated with these auxin gradients. Establishment of these auxin gradients appeared to initiate the polar localization of PIN1 that is responsible for re-establishment of auxin gradients during SAM and somatic embryo formation. Our results suggest that establishment of auxin gradients and the polar distribution of PIN1 are critical for regulation of *WUS* expression during somatic embryogenesis.

## Results

### Genes associated with SAM formation and zygotic embryogenesis are expressed during somatic embryogenesis

**Figure 1:**
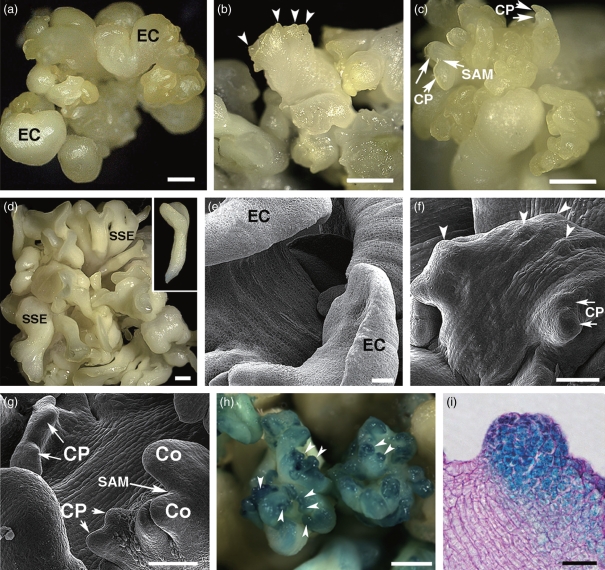
Morphogenesis of Arabidopsis somatic embryos. (a–d) Morphology of somatic embryogenesis by light microscopy. (e–g) Morphology of somatic embryogenesis by scanning electron microscopy. (h, i) Patterns of *pLEC2::GUS* expression by light microscopy. (a, e) Disc-like embryonic calli from PSEs cultured in ECIM for 14 days. (b, f) Somatic pro-embryos on the surfaces of embryonic callus edges (arrowheads) in SEIM at 2 days. (c, g) Two cotyledon primordia and the SAM of an SSE at 4 days. (d) SSEs at 8 days. (h) Strong GUS signals representing *LEC2* transcription accumulate in globular-stage somatic embryos (arrowheads) after 2 days in SEIM. (i) Pattern of *LEC2* expression in an early heart-stage somatic embryo in sectioned tissue. EC, embryonic calli; Co, cotyledon; CP, cotyledon primordium; SAM, shoot apical meristem; SSE, secondary somatic embryo. Scale bars = 1.2 mm (a–d, h), 60 μm (e–g) and 80 μm (i).

**Figure 2:**
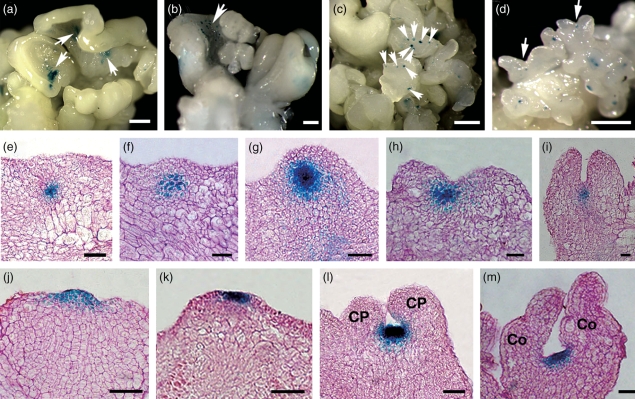
Patterns of *pWUS::GUS* and *pCLV3::GUS* expression during somatic embryogenesis by light microscopy. (a) *pWUS::GUS* signals in a few regions on the inside of disc-like embryonic calli cultured in ECIM for 14 days. (b) Signals on small regions of embryonic callus in SEIM at 24 h. (c) Signals in somatic pro-embryos at 2 days. (d) Signals in the region between two cotyledon primordia at 4 days. (e–i) Sections of embryonic calli in SEIM at 24 and 36 h, and 2, 3 and 4 days, respectively, showing *WUS* expression patterns. (j, k) Patterns of *CLV3* expression at early and late globular stages of embryo development. (l, m) Patterns of *CLV3* expression at late stages of embryo development. Arrows indicate *WUS* expression signals. CP, cotyledon primordium; Co, cotyledons. Scale bars = 1.3 mm (a–d) and 80 μm (e–m).

Previously, it was shown that Arabidopsis somatic embryos were induced by auxin (Ikeda-Iwai *et al.*, 2002). To establish a highly reproducible somatic embryogenesis system, we improved the culture medium for primary somatic embryo induction. Several green primary somatic embryos (PSEs) could be induced from explants cultured on medium containing 2,4-dichlorophenoxyacetic acid (2,4-D) and casein hydrolysate after 10 days. The PSEs were then cultured in liquid medium containing 2,4-D (embryonic callus-inducing medium, ECIM) for 14 days, and disc-like embryonic calli were formed (Figure 1a,e). To induce secondary somatic embryos (SSEs), embryonic calli were transferred to 2,4-D-free liquid medium (somatic embryo-inducing medium, SEIM) for 8 days. At 2 days after transfer to SEIM, many protuberances were produced on the edges of the embryonic callus on the inside surface, suggesting that somatic pro-embryos might be now detectable morphologically (Figure 1b,f). By comparison with the stages of zygotic embryo development as defined by Meinke (1991), it is likely that these somatic pro-embryos could be considered equivalent to the globular stage of developmental potential, although the formation of distinct cell layers was not observed in these somatic pro-embryos (see Figures 1i and 2f–l). At 4 days, cotyledon primordia emerged from the tops of somatic pro-embryos, whose morphology corresponds to that of the heart-stage zygotic embryo, and an SAM formed within the somatic embryo (Figure 1c,g). At 8 days, a large number of SSEs with two distinct cotyledons, a SAM, a hypocotyl and a radicle were obtained from the embryonic callus (Figure 1d). These SSEs had the capacity to grow into adult plants when cultured on solid medium. In addition to the comparatively normal morphology of somatic embryos, a small fraction (approximately 10%) of somatic embryos showed abnormal morphology. Each callus could produce a mean of 42 ± 10 embryos. Thus, the system of the secondary somatic embryogenesis was used for further investigations.

Genes including *LEC1*, *LEC2*, *FUSCA3* (*FUS3*) and *ABA-INSENSITIVE 3* (*ABI3*) play important roles in zygotic embryogenesis (Giraudat *et al.*, 1992; Lotan *et al.*, 1998; Luerßen *et al.*, 1998; Stone *et al.*, 2001). To determine the expression patterns of these genes during somatic embryogenesis, we performed quantitative real-time PCR analyses. The results showed that expression of each of these genes was up-regulated at 2 days after transfer of the embryonic calli to SEIM At 8 days, the levels of *FUS3* and *ABI3* transcripts remained high, but the level of *LEC1* and *LEC2* transcripts had decreased The level of transcription of two other genes, *SHOOT MERISTEMLESS* (*STM*) and *CUP-SHAPED COTYLEDON 2* (*CUC2*), that are involved in SAM and cotyledon formation, respectively, was also examined (Barton and Poethig, 1993; Aida *et al.*, 1997, 1999). The expression pattern of *CUC2* was similar to that of *LEC2*, in that transcripts were more abundant at 2 and 4 days, whereas *STM* showed a different pattern with increased transcription levels achieved at 4 and 8 days, indicating that the SAM has been formed at the heart stage

Further analysis of *LEC2* localization using *pLEC2::*GUS indicated that lower levels of *LEC2* transcripts were present within some regions of embryonic calli cultured on ECIM; however, stronger GUS signals were localized in whole somatic pro-embryos at 2 days in SEIM (Figure 1h,i). Later, GUS signals were detected in the cotyledon primordia and cotyledons (data not shown). These results suggest that expression of genes involved in SAM formation and zygotic embryogenesis is induced during somatic embryogenesis, and that they exhibit largely similar developmental and spatial expression patterns.

### Expression patterns of WUS and CLV3

The *WUS* expression pattern suggests that stem cells in the shoot meristem are specified by an organizing center that is established in the 16-cell pro-embryo (Mayer *et al.*, 1998). By examining its expression pattern during somatic embryogenesis, we were able to assess the spatio-temporally regulated formation of the organizing center. Figure 2(a–i) shows the patterns of *pWUS::*GUS expression during various stages of early somatic embryogenesis. Weak GUS signals were detected in a few regions of embryonic callus grown in ECIM for 14 days (Figure 2a), whereas, after the embryonic calli were transferred to SEIM, stronger GUS signals started to be detected in some small regions of the embryonic callus at around 24 h (Figure 2b). These signals represent the induced *WUS* expression. Later, GUS signals were observed in the regions between the cotyledon primordia and between more developed cotyledons (Figure 2c,d). Furthermore, tissue-section analysis showed that GUS signals were identified in a group of undifferentiated cells located below seven or eight irregular layers of apical cells at around 24 h. Based on our examination, these cells did not show any obvious layered or regular arrangement. Somatic pro-embryos were not observed morphologically at this time, suggesting that these undifferentiated cells with WUS activity might be the initial organizing center (Figure 2e). At later stages of somatic embryo development, *WUS* was constitutively expressed in the organizing center cells.

In shoot meristem development, WUS specifies stem cells that are marked by *CLV3* expression (Weigel and Jürgens, 2002). The expression pattern of *CLV3* appeared to be different from that of *WUS* in several important aspects (Figure 2j–m). Higher levels of *CLV3* transcripts were observed in the cells of whole somatic pro-embryos at the early globular stage (Figure 2j), but *CLV3* signals were later detected in an apical group of cells in somatic pro-embryos and in the region of the newly formed SAM (Figure 2k–m). Another difference was that accumulation of *CLV3* transcripts occurred later than for *WUS* (Figure 2b,e,j). These results indicate that stem cells are formed in the early somatic pro-embryo in a domain that is initially different to that for *WUS*, and that eventually they are positioned in a region that overlays the region of *WUS* expression.

**Figure 3:**
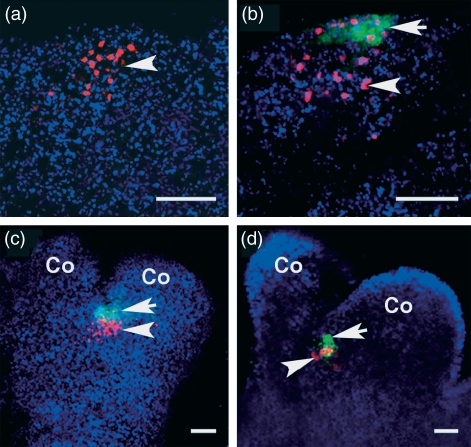
Relative expression domains of *WUS* and *CLV3* by confocal laser scanning microscopy. (a) *WUS* signals (red) in SEIM at 24 h (arrowhead). (b) *CLV3* (green, arrow) and *WUS* (red, arrowhead) signals in the somatic embryo at 2 days. (c, d) Both *CLV3* and *WUS* signals localized within the SAM of the somatic embryo. Co, cotyledon. Scale bars = 60 μm.

To determine the relative expression domains of *WUS* and *CLV3*, we performed an analysis of their co-localization using a *pWUS::DsRED-N7 pCLV3::GFP-ER* marker line. The *WUS* expression domain was located just below the *CLV3* expression domain (Figure 3b–d). *WUS* signals were first detectable in callus grown in SEIM for around 24 h, but *CLV3* signals were not, indicating that *WUS* induction occurs earlier than that of *CLV3* (Figure 3a). Thus, we confirm that both *WUS* and *CLV3* transcription, in non-overlapping domains, are induced following somatic embryo induction, and that formation of an organizing center occurred earlier than formation of stem cells in the SAM and somatic pro-embryo.

### WUS expression patterns at variable levels of auxin

**Figure 4:**
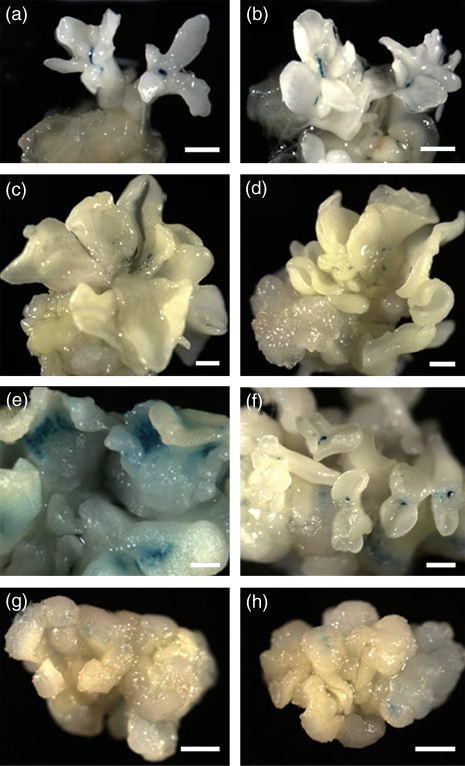
Regulation of *WUS* expression patterns by various levels of auxin in a *pWUS::GUS* marker line. (a, b) PSEs cultured in liquid medium for 22 days. (c–h) PSEs cultured in ECIM for 14 days, and then cultured in SEIM for 8 days (total 22 days). (a, b) Medium without 2,4-D. (c, d) Medium with 1 μm 2,4-D. (e, f) Medium with 9 μm 2,4-D. (g, h) Medium with 18 μm 2,4-D. Scale bars = 1.2 mm.

Induction of *WUS* expression in the embryonic callus required the removal of auxin from the liquid culture medium. However, auxin levels are a prerequisite for the status of embryonic callus. To determine the roles of embryonic callus at various levels of auxin, we analyzed *WUS* expression patterns under various auxin conditions. PSEs were cultured in liquid medium containing various levels of exogenous auxin for 14 days. Then the tissues were cultured in SEIM for 8 days. As shown in Figure 4(a,b), *WUS* transcripts were present in the SAM of PSEs when these PSEs were cultured in SEIM. In medium supplemented with either 1 or 18 μm 2,4-D, embryonic calli with abnormal morphologies were generated from PSEs at 14 days, and SSEs were not induced during further culture for 8 days in SEIM (Figure 4c,d,g,h). Also, *WUS* signals were hardly detected, if at all, in cultured tissues at these levels of auxin. The culture medium used as a control contained 9 μm 2,4-D (see Figure 1). This level of auxin was conducive to correct*WUS* expression during induction (Figure 4e,f). These results suggest that the cell status of embryonic callus is regulated by levels of exogenous auxin in a concentration-dependent manner, and that the levels of exogenous auxin play an essential role in determination of *WUS* expression patterns.

### Establishment of auxin gradients

**Figure 5:**
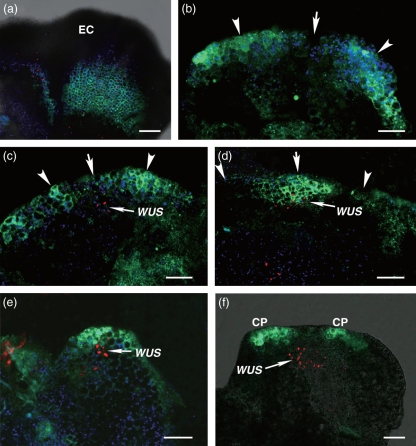
Establishment of dynamic auxin gradients by *DR5rev::GFP* correlated with *WUS* induction by *pWUS::DsRED-N7* within callus. (a) Auxin is distributed in the middle region of embryonic callus (EC), but not in its edge region cultured in ECIM for 14 days. (b) At 16 h after removal of 2,4-D, auxin accumulates in the edge region of embryonic callus in SEIM. The level of auxin in some areas (arrowheads) is higher than in others (arrow). (c) At 24 h after removal of 2,4-D, *WUS* is expressed in the area (arrow) in which auxin accumulates at a low level, and around the area in which it accumulates at a high level. (d, e) Co-localization of *WUS* and auxin signals at early stages of somatic embryogenesis. (f) *WUS* is expressed in the SAM region; whereas auxin accumulates in the cotyledon primordia (CP). Green color indicates *DR5rev::GFP* signals. Red color indicates *WUS* signals. Scale bars = 150 μm (a) and 100 μm (b–f).

**Figure 6:**
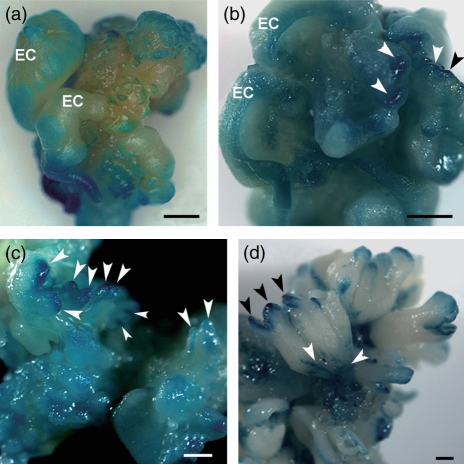
Auxin distribution marked by *DR5::GUS* expression. (a) *DR5::GUS* signals are regionally distributed on embryonic calli cultured in ECIM for 14 days. (b) Strong GUS signals (arrowheads) in somatic pro-embryos are seen on the inside edges of embryonic calli in SEIM at 2 days. (c) GUS signals are detectable in the apical domain of cotyledon primordia (arrowheads). (d) GUS signals accumulate in cotyledons (black arrowhead) and root (white arrowhead) poles of mature somatic embryos. EC, embryonic calli. Scale bars = 0.8 mm (a, b) and 1 mm (c, d).

Correct *WUS* expression was induced by removal of exogenous auxin. We hypothesized that the effect of removal of auxin might function via polar auxin distribution within embryonic callus, something that had not been determined so far. To examine whether *WUS* expression is correlated with the establishment of auxin gradients, we visualized auxin distribution using a *DR5rev::GFP pWUS::DsRED-N7* marker line. After 14 days in ECIM, auxin was distributed in the middle regions of embryonic calli (Figure 5a). Of these calli, a small number (16.8%) contained very weak auxin signals in some edge regions in which correct *WUS* expression and subsequent embryogenesis might occur after removal of auxin (Table 1). Interestingly, strong signals were observed in the edge regions of 76.4% of calli at 16 h after transfer of the embryonic calli to SEIM (Table 1 and Figure 5b). At 24 h after induction, auxin signals were identified in edge regions of 92% calli, indicating that auxin gradients were established in edge regions (Table 1 and Figure 5c). At this time, the induced *WUS* transcripts located in the region below several layers of cells were easily detected in the area of lower auxin levels, and surrounding the area of higher auxin levels. At 36 h, induced *WUS* expression was detectable in more calli (88.5%) than those at 24 h (36.8%) (Table 1 and Figure 5d). Later, auxin signals were re-distributed to the top regions of the somatic pro-embryo and cotyledon primordia (Figure 5e,f). Thus, the results suggest that establishment of auxin gradients is correlated with *WUS* induction within callus, and, after this *WUS* induction, auxin gradients were re-established in the SAM and somatic embryo. In addition, the pattern of *DR5::GUS* expression during somatic embryogenesis was similar to that of *DR5rev::GFP* expression (Figure 6a–d).

**Table 1:** Comparison of detected signals with undetected ones in edge regions within embryonic calli

	Duration in SEIM (h)			
	0	16	24	36
*DR5rev::GFP*	16.8% (16/95)	76.4% (42/55)	92.0% (115/125)	95.0% (76/80)
*pPIN1::PIN1-GFP*	6.7% (5/75)	33.9% (20/59)	80.9% (89/110)	88.9% (80/90)
*pWUS::DsRed-N7*	0 (0/60)	7.3% (4/55)	36.8% (35/95)	88.5% (85/96)

### PIN1 localization

**Figure 7:**
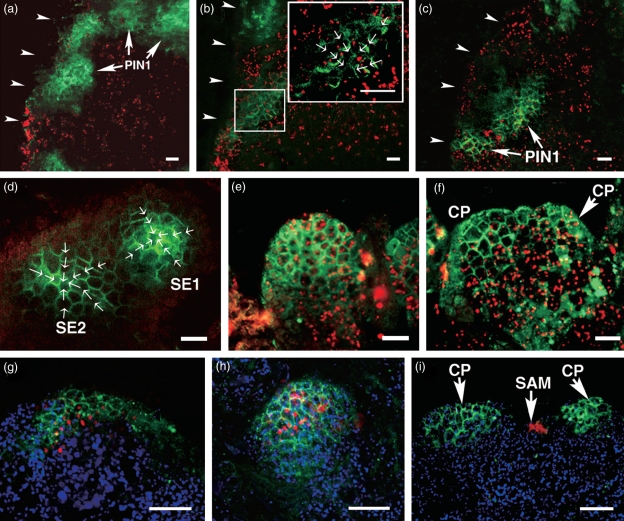
Polar auxin transport indicated by a *pPIN1::PIN1-GFP* reporter. (a) PIN1 polarization does not occur in embryonic calli (EC) cultured in ECIM for 14 days. (b) After 16 h in SEIM, PIN1 localization is polarized to the edge region of embryonic callus. The inset is an enlargement of the box. The arrows show the direction of PIN1 polarization within cells. (c) Increased PIN1 polarization in embryonic callus in SEIM at 24 h. (d, e) Polar localization of PIN1 (green) in early and late globular somatic pro-embryos. The arrows show the direction of PIN1 polarization. (f) Signals in regional cells that will produce cotyledon primordia. (g) At 36 h in SEIM, the signals for *WUS* transcripts and PIN1 are co-localized in the same domain within the callus. (h) Co-localization of *WUS* transcripts and PIN1 in the somatic pro-embryo. (i) *WUS* transcripts are localized in the SAM, whereas PIN1 signals are localized in the cotyledon primordia. SE1 and SE2, regions that will produce somatic embryos; Co, cotyledon; CP, cotyledon primordia; SAM, shoot apical meristem. Arrowheads in (a)–(c) indicate the edge of calli. Green signals are PIN1. Red signals in (a)–(f) and blue signals in (g)–(i) are chlorophyll autofluorescence. The red color in (g)–(i) indicates *WUS* signals. In Scale bars = 50 μm (a–f) and 60 μm (g–i).

Polar localization of PINs is responsible for the directed transport of auxin (Wiśniewska *et al.*, 2006). To determine whether PIN1 proteins are correlated with establishment of auxin gradients, we analyzed the expression patterns of *pPIN1::PIN1-GFP* and *pPIN1::PIN1-GFP pWUS::DsRED-N7* reporters during somatic embryogenesis. Although PIN1 signals were observed in some edge regions of embryonic calli at 14 days, polar localization of PIN1 was clearly detectable in these regions at 16 and 24 h after induction, suggesting that PIN1 proteins are correlated with establishment of auxin gradients in edge regions (Figure 7a–c and Table 1). Further, stronger PIN1 signals were localized in groups of cells located just above the *WUS* expression domain within the same region at around 36 h (Figure 7d,g). At 2 days, PIN1 distribution patterns showed that auxin was transported to the apical cells of somatic pro-embryos (Figure 7e,h). Later, polar localization of PIN1 was identified in cotyledon primordia (Figure 7f,i), and both *PIN1* and *WUS* expression domains became spatially distinct and separated in somatic embryos by the heart stage (Figure 7i). The observed *PIN1* expression patterns imply that this gene plays an important role in polar auxin transport during somatic embryo development.

**Figure 8:**
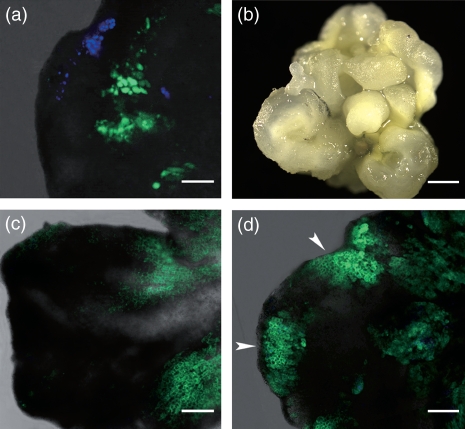
Auxin transport inhibitor affects polar auxin transport and auxin distribution. (a) Following treatment of calli with NPA in ECIM at 12 days, PIN1 polar localization is disrupted, and *WUS* expression is not induced in SEIM at 24 h. Green signals are PIN1 proteins. Blue signals are chlorophyll autoinfluorescence. (b) Somatic embryos are not produced from embryonic calli cultured in SEIM with NPA. (c) Auxin is distributed in the internal middle regions of embryonic callus, but not in edge regions in calli cultured in ECIM containing NPA after 14 days. (d) Strong auxin signals (arrowheads) accumulate regionally at the edge of embryonic callus cultured in SEIM containing NPA for 24 h. Auxin distribution in (c) and (d) is indicated by green signals. Scale bars = 110 μm(a), 0.8 mm (b) and 120 μm (c, d).

To further confirm whether establishment of auxin gradients is involved in polar auxin transport, an auxin transport inhibitor, N-1-naphthylphthalamic acid (NPA, 10 μm) was added to ECIM at 12 days. Treatment of the embryonic calli with NPA resulted in the disruption of PIN1 polar distribution at 24 h after induction, and regeneration of somatic embryos was completely inhibited (Figure 8a,b). Also, *WUS* induction did not occur within the callus, confirming that polar auxin transport has an essential role in SAM formation and somatic embryo induction (Figures 8a). Moreover, the auxin signals were strong in some edge regions of embryonic calli at 24 h after induction, and distribution of signals was confined to small regions compared with the untreated calli at the same induction time point (Figures 5c and 8c,d). Thus it is likely that auxin gradients are mediated by polar auxin transport during somatic embryo induction.

### *WUS, PIN1* and *TIR1* functional analysis

**Figure 9:**
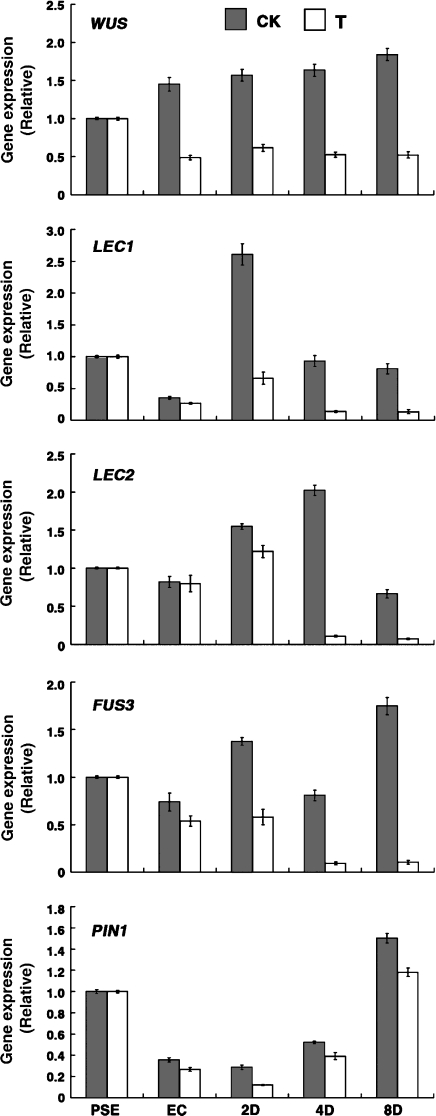
Analysis of gene expression in cultured tissues carrying antisense *WUS* cDNA. Quantitative real-time PCR analysis showing expression patterns of *WUS*, *LEC1*, *LEC2*, *FUS3* and *PIN1*. CK, explants cultured in ECIM and SEIM without the estrogen inducer; T, explants were cultured in ECIM and SEIM containing the estrogen inducer for 14 days and 8 days, respectively.

**Figure 10:**
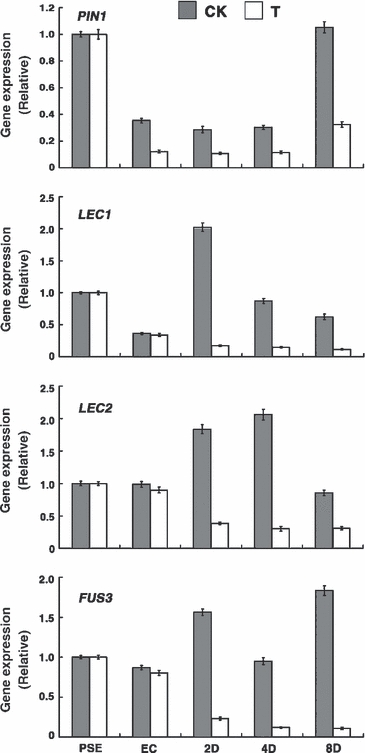
Analysis of gene expression in cultured tissues carrying antisense *PIN1* cDNA. Quantitative real-time PCR analysis showing expression patterns of *PIN1*, *LEC1*, *LEC2* and *FUS3*. CK, explants cultured in ECIM and SEIM without the estrogen inducer; T, explants were cultured in ECIM and SEIM containing the estrogen inducer for 14 days and 8 days, respectively.

**Figure 11:**
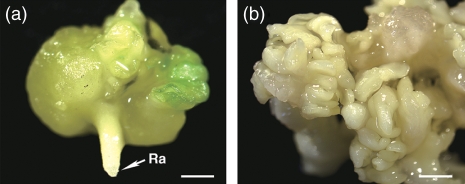
Functional analysis of both *PIN1* and *TIR1* during somatic embryogenesis. (a) Primary somatic embryos cannot be induced from explants of the *pin1* mutant, although a single radicle (Ra) is visible. (b) Mutation in *TIR1* results in severely abnormal somatic embryos. Scale bars = 0.4 mm (a) and 1.2 mm (b).

To determine the roles of *WUS* and *PIN1* during somatic embryogenesis, we constructed vectors carrying either antisense *WUS* or *PIN1* genes driven by an estrogen receptor-based transactivator XVE (Zuo *et al.*, 2000), and transferred these constructs to Arabidopsis plants. PSEs were cultured in ECIM with estrogen for 14 days, and then cultured in SEIM with estrogen for another 8 days. By comparison with untreated cultured tissues using estrogen as a control, only 8.9% embryonic calli carrying the antisense *WUS* cDNA produced abnormal somatic embryos, and each embryonic callus generated only 3.3 ± 1.5 abnormal somatic embryos over the full 22 days of culture (Table 2). The quantitative real-time PCR analysis confirmed that levels of *WUS* transcripts were substantially decreased in cultured tissues carrying antisense *WUS* cDNA after estrogen was supplied, compared with non-induced tissues (Figure 9). Moreover, transcript levels of *LEC1*, *LEC2*, *FUS3* and *PIN1* were decreased in cultured transgenic tissues (Figure 9). We also examined *PIN1* function using the same strategy. Inhibition of *PIN1* expression also resulted in disruption of somatic embryogenesis and decreased transcript levels of genes involved in embryogenesis (Table 2 and Figure 10). Heterozygous *pin1*–*7* mutants in the Columbia background were used as explants for somatic embryo induction, because its homozygous mutants cannot produce seeds (Smith *et al.*, 2006). As expected, almost a quarter of the explants could not produce PSEs (Figure 11a). The results suggest that both *WUS* and *PIN1* are required for somatic embryo formation.

**Table 2:** Comparison of regeneration frequency of somatic embryos between wild-type, *pER8-WUS antisense* and *pER8-PIN1 antisense* explants

	Wild-type		*pER8-WUS antisense*		*pER8-PIN1 antisense*	
Days^a^	22	*	22	*	22	*
Ratio^b^	77.8%	94.5%	8.9%	90.1%	9.5%	85.5%
Number^c^	24.1 ± 4.6	45.5 ± 7.8	3.3 ± 1.5	37.5 ± 3.1	5.0 ± 1.2	33.5 ± 4.4
Status^d^	Normal	Normal	Abnormal	Normal	Abnormal	Normal

a Duration of application of β-estradiol (days). The asterisk indicates that estradiol was not applied.

b Proportion of embryonic calli that produced somatic embryos.

c Number of somatic embryos produced per embryonic callus (mean ± SE, *n*=90).

d Developmental status of somatic embryos.

TIR1 acts as an auxin receptor in Arabidopsis. It is an integral component of the SKP1/Cullin/F-box protein (SCF)^TIR1^ complex and mediates auxin signaling (Lau *et al.*, 2008). The *tir1* mutant in the Columbia background is deficient in hypocotyl elongation and lateral root formation (Ruegger *et al.*, 1998). Although the *tir1* mutant produces normal seeds in plants, it generated only severely abnormal SSEs after induction in these experiments (Figure 11b). Therefore, a functional auxin signaling mechanism that involves TIR1 mediation is necessary for normal somatic embryogenesis.

## Discussion

### Characterization of somatic embryogenesis

During somatic embryogenesis, the developmental process from the globular stage to the torpedo stage is similar to that of zygotic embryogenesis (Meinke, 1991; Zimmerman, 1993). As for the morphological similarity of characters between embryo types, the expression of important regulatory genes, such as *LEC1*, *LEC2*, *FUS3* and *ABI3*, that is observed in zygotic embryogenesis, was also seen in somatic embryogenesis, and their transcript levels increased following induction treatment Therefore, it is reasonable to hypothesize that these genes, which act as critical genes during zygotic embryogenesis, might be necessary for somatic embryogenesis.

Although the somatic and zygotic embryos are similar developmentally and morphologically, the anatomical structure of the somatic embryo is different from that of the zygotic embryo. In Arabidopsis, the zygotic embryos at the globular and heart stages comprise obviously identifiable layers and regions, reflecting zygotic embryo patterns established at these stages (Meinke, 1991; Weigel and Jürgens, 2002). The *Arabidopsis thaliana MERISTEM LAYER 1* (*AtML1*) gene is expressed in the protoderm from the 16-cell pro-embryo to the heart-stage embryo, and, later, only in the L1 layer of the SAM. This unusual pattern of expression suggests that this gene plays a regulatory role in meristem organization, perhaps by specifying the surface layer of the SAM early in zygotic embryogenesis, or later by directing the differentiation of L1-derived cells (Lu *et al.*, 1996). In contrast, it is likely that the protoderm can be identified in cells of the somatic embryo after the heart stage of embryo development (Figure 2i,m), suggesting that the different patterns of protoderm formation between zygotic embryogenesis and somatic embryogenesis may be regulated by *AtLM1* expression.

The first shoot meristem marker, i.e. *WUS*, is initiated in only four inner cells of the 16-cell zygotic pro-embryo (Weigel and Jürgens, 2002), whereas in somatic embryogenesis *WUS* was activated in a group of cells (more than four) after transfer of embryonic callus to SEIM, and its induction was essential for somatic embryogenesis (Figure 2e,f and Table 2). These cells containing *WUS* transcripts did not show any regular pattern or arrangement. Another important finding is that more *WUS* transcripts were detectable at 24 h than at 16 h after induction, but the somatic pro-embryo could not be identified morphologically or molecularly until later, indicating that *WUS* induction occurs earlier in somatic embryogenesis than in zygotic embryogenesis.

During early somatic embryogenesis, auxin is transported to an apical cell, and the newly established auxin gradient is responsible for the subsequent formation of apical embryo structures. Next an auxin gradient is established in basal cells of the globular embryo. PIN1 is involved in polar auxin transport in both the apical and basal root poles (Friml *et al.*, 2003). Following *WUS* induction during somatic embryogenesis, auxin accumulation occurred first in apical cells of the pro-embryo and later in cotyledon primordia, and finally in the radicle (Figures 5e,f and 6c,d). PIN1 localization indicated polar auxin transport to apical cells of the pro-embryo and apical cells of the cotyledon primordia. The early events shared between somatic embryogenesis and zygotic embryogenesis are different in several respects, not only morphologically but also molecularly. Specific characteristics of somatic embryogenesis might be due to the origin of the somatic embryo from embryonic callus, whereas the zygotic embryo is derived from a highly differentiated and polarized single-cell zygote wholly contained within the embryo sac.

### Established auxin gradients are correlated with induced WUS expression

The presence of auxin is required for somatic embryo induction. In carrot cell cultures, induction of somatic embryos occurred after culture with exogenous auxin and its removal (Schiavone and Cooke, 1987; Zimmerman, 1993). In Arabidopsis, exogenous auxin is also a prerequisite for somatic embryo induction (Ikeda-Iwai *et al.*, 2002). In addition, previous studies have shown PIN2 polarity changes in response to gravity stimulation (Friml *et al.*, 2003; Abas *et al.*, 2006), which could be considered a type of auxin removal. We hypothesize that removal of auxin from the culture medium may be a stress factor that causes polar auxin distribution in specific regions that produce somatic embryos. Following auxin removal from the culture medium, auxin gradients were established in some edge regions of embryonic callus at around 24 h, indicating that removal of auxin induced the establishment of auxin gradients in these regions (Figure 5a–c). Induced *WUS* transcripts were first detectable in these regions in areas of low auxin levels, and around areas of higher auxin levels. In support of these results, it has been reported previously that the shoot meristem in Arabidopisis is initiated in areas of low auxin concentration during shoot regeneration from callus (Gordon *et al.*, 2007). In this study, following *WUS* induction, auxin gradients were re-established in the SAM region and then in the cotyledon primordia, implying that establishment of auxin gradients within callus and pro-embryo tissues plays an important role in SAM formation and somatic embryo development. Additionally, the *tir1* mutants generated severely abnormal somatic embryo phenotypes (Figure 11b), suggesting that auxin signaling mediated by *TIR1* is required for somatic embryogenesis.

### Auxin gradients are involved in PIN1 localization

The direction of auxin flow is determined by the asymmetric cellular localization of auxin efflux carriers (Raven, 1975). In this study, the establishment of auxin gradients in embryonic callus was coincident with the marked polar distribution of PIN1, which was observed in edge regions of embryonic callus at 16 and 24 h (Figures 5a–c and 7a–c). This is consistent with a model indicating that auxin mediates PIN localization through a positive feedback mechanism to maintain the polarized localization of PINs (Gao *et al.*, 2008). Following *WUS* induction in areas of low auxin and around areas of high auxin, PIN1 localization was observed in a group of apical cells that are localized directly above the *WUS* expression domain at around 36 h (Figures 5c,d and 7d,g). It is likely that auxin was transported from areas of high concentration areas to areas of low concentration, with polar transport mediated by PIN proteins. This polar auxin transport resulted in re-establishment of auxin gradients in the SAM and somatic pro-embryo (Figure 5c–e). This mechanism is supported by the fact that treatment of embryonic calli with NPA caused accumulation of higher levels of auxin in smaller localized regions of embryonic calli than in untreated calli at 24 h after induction (Figures 5c and 8d). Previously, it has been shown that PIN1 is required for the initiation and maintenance of auxin gradients within plant tissues (Friml *et al.*, 2003; Heisler *et al.*, 2005). Thus, establishment of auxin gradients is a self-polarizing signal that may activate the polar localization of PINs, such as PIN1, within callus (Gao *et al.*, 2008), and then the polarized PIN1 proteins cause localized auxin accumulation in the SAM and pro-embryo following *WUS* induction.

During shoot regeneration, the mean number of shoots in the *pin1–4* mutant decreased to approximately 20% of that in wild-type (Gordon *et al.*, 2007). In the present study, regeneration of somatic embryos was severely inhibited in the *pin1–7* mutant and in transgenic plants containing antisense *PIN1* cDNA (Figure 11a and Table 2). Moreover, in cultured tissues containing antisense *PIN1* cDNA, transcription of several genes was decreased after induction compared with control tissues (Figure 10). Experiments using the auxin transport inhibitor NPA found that its use did not result in any induction of *WUS* expression and somatic embryogenesis. In conclusion, PIN1 mediation of polar auxin transport is essential for formation of the stem cell organizing center, SAM formation, and somatic embryogenesis.

## Experimental procedures

### Plant materials

Arabidopsis plants used in this study were of the Columbia ecotype. The *pLEC2::GUS* reporter lines were a gift from Dr F. Parcy (Institut des Sciences du Végétal, Centre National de la Recherche Scientifique, Gif sur-Yvette,, France). The *pCLV3::GUS* marker line was kindly provided by Dr R. Simon (Institut für Entwicklungsbiologie der Universität zu Köln, Germany). The translational protein fusion construct *pPIN1::PIN1-GFP* was obtained from Dr E.M. Meyerowitz (Division of Biology, California Institute of Technology, Pasadena, USA) and was transformed into Arabidopsis. The transgenic plants were crossed with plants harboring the *pWUS::DsRED-N7* construct (provided by Dr E.M. Meyerowitz). The *pWUS::DsRED-N7 pCLV3::GFP-ER* double reporter lines were also provided by Dr Meyerowitz. The *DR5::GUS* lines (provided by Dr H.W. Xue, Institute of Plant Physiology and Ecology, Chinese Academy of Sciences, Shanghai) and the *DR5rev::GFP* lines (provided by Dr J. Friml, Zentrum für Molekularbiologie der Pflanzen, Universität Tübingen, Germany), were used separately to visualize auxin distribution during somatic embryogenesis. The *DR5rev::GFP* lines were then crossed with *pWUS::DsRED-N7* plants. The estradiol-inducible XVE binary vector was kindly provided by Dr W.C. Yang (Institute of Genetics and Developmental Biology, Chinese Academy of Sciences, Beijing).

### Plant growth conditions, *in vitro* culture, and somatic embryogenesis

Arabidopsis surface-sterilized seeds were plated on germination medium (half-strength MS, Murashige and Skoog, 1962). The plates were kept at 4°C in darkness for 2 days to overcome dormancy, and then transferred to a growth room maintained at 20–22°C with a 16 h light/8 h dark cycle for 7 days. Young seedlings were transplanted into vermiculite and grown under the conditions as described above until harvesting of siliques.

Somatic embryo induction was performed using a modified method based on that described by Ikeda-Iwai *et al.* (2002). Immature zygotic embryos were collected as explants and used to obtain PSEs. To obtain greater numbers of PSEs, casein hydrolysate (500 mg l^−1^, Sigma, http://www.sigmaaldrich.com/) was added to agar-solidified B5 medium. The PSEs were transferred into liquid B5 medium containing 9.0 μm 2,4-D (ECIM) and pre-cultured for 14 days. Then, embryonic calli were cultured in auxin-free liquid B5 medium (SEIM) to induce SSEs.

### Total RNA isolation and quantitative real-time PCR analysis

Total RNAs were isolated from zygotic embryos, PSEs and embryonic calli cultured in ECIM for 14 days, and somatic embryos in SEIM at 2, 4 and 8 days. TRIzol reagent (Invitrogen, http://www.invitrogen.com/) was used for quantitative real-time PCR. Total RNAs were treated with DNase I (RNase-free, Promega, http://www.promega.com/) to remove any DNA contamination. The cDNAs were synthesized using the oligo(dT) primer 5′-GACTCGAGTCGACATCGATTTTTTTTTTTTTTTTT-3′ and the M-MLV kit according to the manufacturer’s instructions (Promega).

All primers used for quantitative real-time PCR are shown in The quantitative real-time PCR reactions were performed on each cDNA dilution using SYBR Green Master mix with Chromo4 according to the manufacturer’s protocol (Bio-Rad, http://www.bio-rad.com/). The PCR cycles were as follows: one cycle of 30 sec at 95°C, followed by 45 cycles each of 5 sec at 95°C, 10 sec at 58–63°C, and 15 sec at 72°C. Measurements were carried out in triplicate. The results were analyzed using the comparative *C*_T_ method, and means and standard deviations were calculated.

### *pWUS::GUS* construction

To construct *pWUS::GUS*, a 2.4 kb DNA promoter fragment upstream of the transcriptional start site of the *WUS* gene (At2g17950) was amplified and then cloned into the pBI121 vector. Transgenic plants were screened for kanamycin resistance (50 mg l^−1^, Promega) and used in further experiments.

### β-GUS assays

GUS histochemical staining assay was performed as described by Sieburth and Meyerowitz (1997). To investigate reporter expression pattern, tissues showing GUS staining were fixed, cleared and embedded in paraffin (Sigma). At least 30 embedded tissues for each sample were sectioned. To display clearly the outline of cells, slides were stained with 100 mg l^−1^ ruthenium red (Sigma-Aldrich) in distilled water for 3–5 min. Then the stained sections were photographed using an Olympus BH-2 microscope equipped with an Olympus DP12 digital camera (http://www.olympus-global.com/).

### Antisense *WUS* or *PIN1* cDNA plasmid constructions

To determine the *WUS* and *PIN1* antisense expression patterns during somatic embryo induction, a 874 bp cDNA fragment of the *WUS* (At2g17950) coding region was amplified using primers 5′-GTACTAGTGCCACAGCATCAGCATCATCAT-3′ (forward) and 5′-GTCTCGAGCTAGTTCAGACGTAGCTCAAGAG-3′ (reverse). A 922 bp cDNA fragment of the *PIN1* (At1g73590) coding region was amplified using primers 5′-AGCTTTTGATCTCCGAGCAGTT-3′ (forward) and 5′-ATGAGTCTTGTCATCACACTTGTT-3′ (reverse). *Spe*I and *Xho*I sites were introduced into the 5′ end of both primers. *WUS* and *PIN1* antisense cDNAs were then cloned into the estradiol-inducible XVE binary vector (Zuo *et al.*, 2000), and transformed into Arabidopsis plants. To monitor estradiol-induced production of cDNA-encoded transcripts, semi-quantitative RT-PCR was performed (data not shown).

### Chemicals and induction

To induce transcription of inserted cDNAs, the PSEs were cultured in ECIM with 10 μm estradiol (prepared in DMSO as 10 mm stock; Sigma) for 14 days. Then the cultured tissues were transferred to SEIM with 10 μm estradiol for another 8 days. Estradiol was added every 2 days. Total RNA was isolated from PSEs and embryonic calli in ECIM with estradiol at 14 days, and from somatic embryos in SEIM with estradiol at 2, 4 and 8 days. These tissues were used for quantitative real-time PCR to detect the expression of *WUS*, *PIN1* and several other genes.

### Imaging conditions

The morphology of somatic embryos was examined and photographed using an Olympus JM dissecting microscope. For scanning electron microscopy, samples were treated and micrographs were taken as described by Li *et al.* (2002).

To investigate the expression pattern of reporter genes, samples were observed using a Zeiss 510 Meta laser scanning confocal microscope (http://www.zeiss.com/) with a 20× air objective, 40× oil dipping lens, or a 63× oil dipping lens. Sets of filters used to visualize GFP alone, or GFP and DsRED together, were selected as described previously by Heisler *et al.* (2005). Zeiss LSM software was used to analyze the confocal imaging. For each gene marker line at the various culture stages, at least 30 samples were imaged to confirm the expression pattern of the marker in each stage.

## SUPPORTING INFORMATION

**Figure S1.** The qRT-PCR analysis shows expression patterns of several genes involved in embryo development during somatic embryogenesis.

**Figure S2**. The transcripts of *WUS* in proembryo were detected by in situ hybridization.

**Table S1.** Primers sequences used for qRT-PCR analysis of gene expression during somatic embryogenesis shown in Figures 9, 10, and S1.

## References

[b1] Abas L, Benjamins R, Malenica N, Paciorek T, Wiśniewska J, Moulinier-Anzola JC, Sieberer T, Friml J, Luschnig C (2006). Intracellular trafficking and proteolysis of the Arabidopsis auxin-efflux facilitator PIN2 are involved in root gravitropism. Nat. Cell Biol..

[b2] Aida M, Ishida T, Fukaki H, Fujisawa H, Tasaka M (1997). Genes involved in organ separation in Arabidopsis. An analysis of the cup-shaped cotyledon mutant. Plant Cell.

[b3] Aida M, Ishida T, Tasaka M (1999). Shoot apical meristem and cotyledon formation during Arabidopsis embryogenesis, interaction among the *CUP-SHAPED COTYLEDON* and *SHOOT MERISTEMLESS* genes. Development.

[b4] Barton MK, Poethig RS (1993). Formation of the shoot apical meristem in *Arabidopsis thaliana*, an analysis of development in the wild type and in the *shoot meristemless* mutant. Development.

[b5] Birnbaum KD, Alvarado AS (2008). Slicing across kingdoms: regeneration in plants and animals. Cell.

[b6] Brand U, Fletcher JC, Hobe M, Meyerowitz EM, Simon R (2000). Dependence of stem cell fate in *Arabidopsis* on a feedback loop regulated by *CLV3* activity. Science.

[b7] Fletcher LC, Brand U, Running MP, Simon R, Meyerowitz EM (1999). Signaling of cell fate decisions by *CLAVATA3* in *Arabidopsis* shoot meristems. Science.

[b8] Friml J, Vieten A, Sauer M, Weijers D, Schwarz H, Hamann T, Offringa R, Jürgens G (2003). Efflux-dependent auxin gradients establish the apical–basal axis of *Arabidopsis*. Nature.

[b9] Gaj MD, Zhang SB, Harada JJ, Lemaux PG (2005). Leafy cotyledon genes are essential for induction of somatic embryogenesis of *Arabidopsis*. Planta.

[b10] Gallois JL, Nora FR, Mizukami Y, Sablowski R (2004). WUSCHEL induces shoot stem cell activity and developmental plasticity in the root meristem. Genes Dev..

[b11] Gao XW, Nagawa S, Wang GX, Yang ZB (2008). Cell polarity signaling: focus on polar auxin transport. Mol. Plant.

[b12] Giraudat J, Hauge BM, Valon C, Smalle J, Parcy F, Goodman HM (1992). Isolation of the *Arabidopsis ABI3* gene by positional cloning. Plant Cell.

[b13] Gordon SP, Heisler MG, Reddy GV, Ohno C, Das P, Meyerowitz EM (2007). Pattern formation during *de novo* assembly of the *Arabidopsis* shoot meristem. Development.

[b14] Heisler MG, Ohno C, Das P, Sieber P, Reddy GV, Long JA, Meyerowitz EM (2005). Patterns of auxin transport and gene expression during primordium development revealed by live imaging of the *Arabidopsis* inflorescence meristem. Curr. Biol..

[b15] Ikeda-Iwai M, Satoh S, Kamada H (2002). Establishment of a reproducible tissue culture system for the induction of *Arabidopsis* somatic embryos. J. Exp. Bot..

[b16] Lau S, Jürgens G, De Smet I (2008). The evolving complexity of the auxin pathway. Plant Cell.

[b17] Laux T, Mayer KFX, Berger J, Jürgens G (1996). The *WUSCHEL* gene is required for shoot and floral meristem integrity in *Arabidopsis*. Development.

[b18] Lenhard M, Laux T (2003). Stem cell homeostasis in the *Arabidopsis* shoot meristem is regulated by intercellular movement of CLAVATA3 and its sequestration by CLAVATA1. Development.

[b19] Li QZ, Li XG, Bai SN, Lu WL, Zhang XS (2002). Isolation of *HAG1* and its regulation by plant hormones during *in vitro* floral organogenesis in *Hyacinthus orientalis* L. Planta.

[b20] Liu CM, Xu ZH, Chua NH (1993). Auxin polar transport is essential for the establishment of bilateral symmetry during early plant embryogenesis. Plant Cell.

[b21] Lotan T, Ohto M, Yee KM, West MAL, Lo R, Kwong RW, Yamagishi K, Fischer RL, Goldberg RB, Harada JJ (1998). *Arabidopsis* LEAFY COTYLEDON1 is sufficient to induce embryo development in vegetative cells. Cell.

[b22] Lu P, Porat R, Nadeau JA, O’Neill SD (1996). Identification of a meristem L1 layer-specific gene in Arabidopsis that is expressed during embryonic pattern formation and defines a new class of homeobox genes. Plant Cell.

[b23] Luerßen H, Kirik V, Herrmann P, Misera S (1998). *FUSCA3* encodes a protein with a conserved VP1/ABI3-like B3 domain which is of functional importance for the regulation of seed maturation in *Arabidopsis thaliana*. Plant J..

[b24] Mayer KFX, Schoof H, Haecker A, Lenhard M, Jürgens G, Laux T (1998). Role of *WUSCHEL* in regulating stem cell fate in the *Arabidopsis* shoot meristem. Cell.

[b25] Meinke DW (1991). Perspectives on genetic analysis of plant embryogenesis. Plant Cell.

[b26] Murashige T, Skoog F (1962). A revised medium for rapid growth and bio-assays with tobacco tissue cultures. Physiol. Plant..

[b27] Okada K, Ueda J, Komaki MK, Bell CJ, Shimura Y (1991). Requirement of the auxin polar transport system in early stages of *Arabidopsis* floral bud formation. Plant Cell.

[b28] Raven J (1975). Transport of indolacetic acid in plant cells in relation to pH and electrical potential gradients, and its significance for polar IAA transport. New Phytol..

[b29] Rojo E, Sharma VK, Kovaleva V, Raikhel NV, Fletcher JC (2002). CLV3 is localized to the extracellular space, where it activates the Arabidopsis CLAVATA stem cell signaling pathway. Plant Cell.

[b30] Ruegger M, Dewey E, Gray WM, Hobbie L, Turner J, Estelle M (1998). The TIR1 protein of *Arabidopsis* functions in auxin response and is related to human SKP2 and yeast Grr1p. Genes Dev..

[b31] Scheres B (2007). Stem-cell niches: nursery rhymes across kingdoms. Nat. Rev. Mol. Cell Biol..

[b32] Schiavone FM, Cooke TJ (1987). Unusual patterns of somatic embryogenesis in the domesticated carrot, developmental effects of exogenous auxins and auxin transport inhibitors. Cell Differ..

[b33] Schoof H, Lenhard M, Haecker A, Mayer KFX, Jürgens G, Laux T (2000). The stem cell population of *Arabidopsis* shoot meristems is maintained by a regulatory loop between the *CLAVATA* and *WUSCHEL* genes. Cell.

[b34] Sieburth LE, Meyerowitz EM (1997). Molecular dissection of the *AGAMOUS* control region shows that *cis* elements for spatial regulation are located intragenically. Plant Cell.

[b35] Smith RS, Guyomarc’h S, Mandel T, Reinhardt D, Kuhlemeier C, Prusinkiewicz P (2006). A plausible model of phyllotaxis. Proc. Natl Acad. Sci. USA.

[b36] Steward FC, Mapes MO, Smith J (1958). Growth and organized development of cultured cells. I. Growth and division of freely suspended cells. Am. J. Bot..

[b37] Stone SL, Kwong LW, Yee KM, Pelletier J, Lepiniec L, Fischer RL, Goldberg RB, Harada JJ (2001). *LEAFY COTYLEDON2* encodes a B3 domain transcription factor that induces embryo development. Proc. Natl Acad. Sci. USA.

[b38] Weigel D, Jürgens G (2002). Stem cells that make stems. Nature.

[b39] Wiśniewska J, Xu J, Seifertová D, Brewer PB, Růžička K, Blilou I, Rouquié D, Benková E, Scheres B, Friml J (2006). Polar PIN localization directs auxin flow in plants. Science.

[b40] Zimmerman JL (1993). Somatic embryogenesis: a model for early development in higher plants. Plant Cell.

[b41] Zuo JR, Niu QW, Chua NH (2000). An estrogen receptor-based transactivator XVE mediates highly inducible gene expression in transgenic plants. Plant J..

