# Supplementary figures and images for: Auxin-induced WUS expression is essential for embryonic stem cell renewal during somatic embryogenesis in Arabidopsis
Source: Plant J. 2009 May 7;59(3):448–60. doi: 10.1111/j.1365-313X.2009.03880.x (PMC2788036; doi:10.1111/j.1365-313X.2009.03880.x)

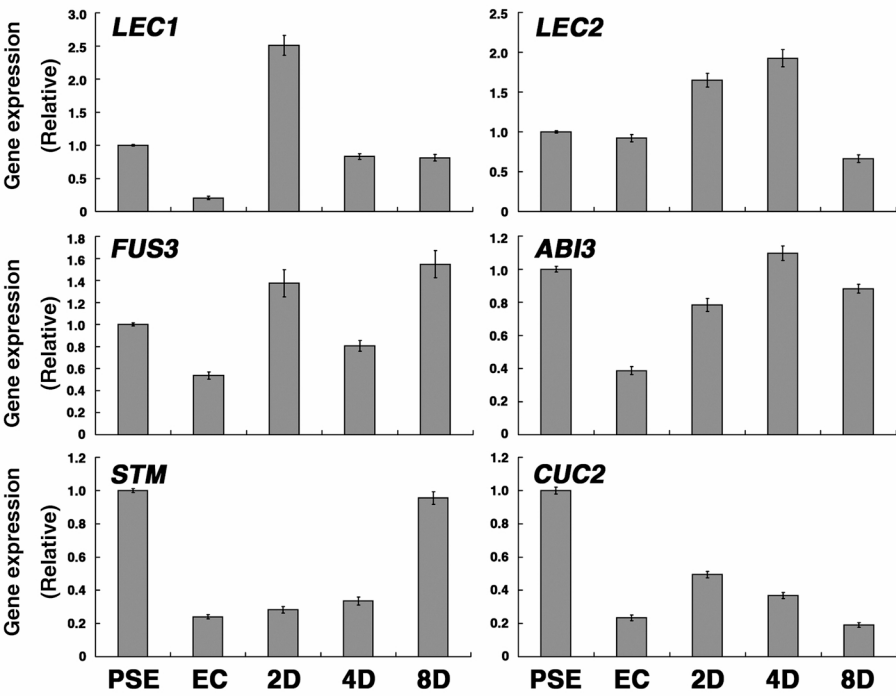

Supplement: Supplementary file 1 [file tpj0059-0448-SD1.pdf]

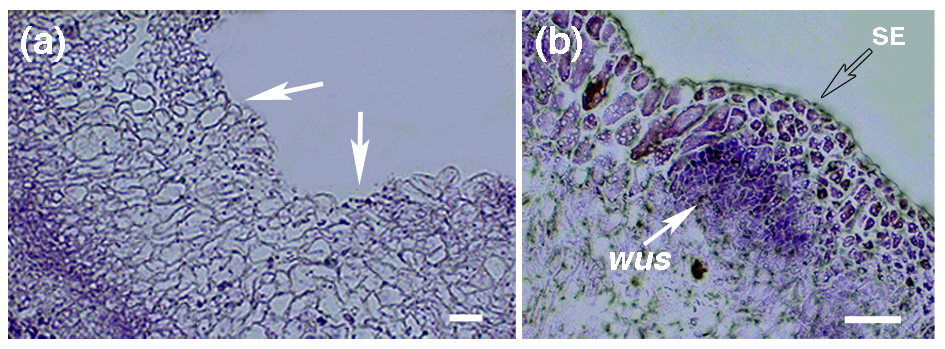

Supplement: Supplementary file 2 [file tpj0059-0448-SD2.tif]
