# Supplementary material for: Auxin-induced WUS expression is essential for embryonic stem cell renewal during somatic embryogenesis in Arabidopsis
Source: Plant J. 2009 May 7;59(3):448–60. doi: 10.1111/j.1365-313X.2009.03880.x (PMC2788036; doi:10.1111/j.1365-313X.2009.03880.x)
Supplement: Supplementary file 3 [file tpj0059-0448-SD3.doc]

**Table S1.**

| Genes | Primers | Primer sequence (5´→3´) |
| --- | --- | --- |
| *TUBULIN2* | Forward | ATCCGTGAAGAGTACCCAGAT |
|  | Reverse | AAGAACCATGCACTCATCAGC |
| *LEC1* | Forward | ACCAGCTCAGTCGTAGTAGCC |
|  | Reverse | GTGAGACGGTAAGGTTTTACGCATGAT |
| *LEC2* | Forward | CTCTCTCTCTCTCCGGGAAA |
|  | Reverse | CCATCTGCTCCACCGGGTAT |
| *FUS3* | Forward | CTCCGACGTATGATACTCCCGAAG |
|  | Reverse | CGCCTGTGTTTTCTAGCACGTACATT |
| *ABI3* | Forward | TATCTTCAGCCGTCTCAAC |
|  | Reverse | CATCTCTTCTATCTTTGTTTCTAC |
| *WUS* | Forward | CCAGCTTCAATAACGGGAATTTAAATCATGCA |
|  | Reverse | TCATGTAGCCATTAGAAGCATTAACAACACCACAT |
| *PIN1* | Forward | TGTTACTGTTCGTCGTTCTAATGC |
|  | Reverse | ACCACCAGAAGCCATCATCG |
| *CUC2* | Forward | CAGCCGTAGCACCAACACAA |
|  | Reverse | GTCTAAGCCCAAGGCCGTAGTA |
| *STM* | Forward | GTCAAGGCCAAGATCATGGCT |
|  | Reverse | TGGTGCTCCAACCTTCTGACA |
